# Microstructure Evolution of TB18 Alloy after Thermal Treatment and the Effect of Recrystallization Texture on Mechanical Properties

**DOI:** 10.3390/ma17122828

**Published:** 2024-06-10

**Authors:** Wei Xiang, Qineng Li, Feng Zhang, Yuan Fan, Wuhua Yuan

**Affiliations:** 1 College of Materials Science and Engineering, Hunan University, Changsha 410082, China; xiangwei03300121@163.com (W.X.); 17886978856@163.com (Q.L.); zf825@foxmail.com (F.Z.); 17712824140@163.com (Y.F.); 2 China National Erzhong Group Deyang Wanhang Die Forging Co., Ltd., Deyang 618013, China

**Keywords:** Ti alloys, hot deformation, static recrystallization, texture evolution, mechanical properties

## Abstract

In industrial production, the deformation inhomogeneity after metal forging affects the mechanical properties of various parts of the forgings. The question of whether the organization and mechanical properties of β-titanium alloy can be improved by controlling the amount of forging deformation needs to be answered. Therefore, in this paper, a new sub-stable β-Ti alloy TB 18 (Ti-5.3Cr-4.9Mo4.9V-4.3Al-0.9Nb-0.3Fe) was subjected to three different levels of deformation, as well as solid solution-aging treatments, and the variation rules of microstructure and mechanical properties were investigated. During the solid solution process, the texture evolution pattern of the TB18 alloy at low deformation (20–40%) is mainly rotational cubic texture deviated into α-fiber texture; at high deformation (60%), the main components of the deformed texture are α-fiber texture with a specific orientation of (114)<1$\overset{-}{13}$3>. After subsequent static recrystallization, the α-fiber texture is deviated to an α*-fiber texture, while the specific orientation (114)<1$\overset{-}{13}$3> can still be inherited as a major component of the recrystallized texture. The plasticity of the alloy in the normal direction (ND) after the solid solution is influenced by the existence of the <110>//ND texture, and the plasticity of the alloy in the ND direction after aging is determined by a combination of the volume fraction of the <110>//ND texture in the matrix phase and the volume fraction of [11$\overset{-}{2}$0]_α_//ND in the α phase. The results show that it is feasible to change the characteristics of the recrystallization texture of TB18 by controlling the deformation level of hot forging, thus realizing the modulation of the mechanical properties.

## 1. Introduction

Due to the high specific strength, fatigue resistance, corrosion resistance, and better resistance to crack extension [1,2], titanium and its alloys are often used in the fields of aircraft wing and fuselage frames, girders, bulkheads, rivets and landing gears, biomedical joints, bone plates, dental implants and stents, and structural and absorptive materials for slow neutron reactors in nuclear power plants [3,4,5]. The β-Ti alloys have higher room temperature plasticity and mechanical strength compared with single-phase (α) and two-phase (α + β) alloys. In the meantime, β-Ti alloys display a variety of microstructural features, such as combinations of α (equiaxed/lamellar) and β phases, which are largely dependent on the thermoplastic mechanical processing (TMP) conditions of α in the transformed β matrix [6]. However, isometric α with transformed β shows higher ductility and thermal stability but low fracture toughness [7]. On the other hand, the lamellar structure of the α-phase in the transformed β-phase shows high impact and fracture toughness but with a severe loss of plasticity.

In addition to the precipitation of the α-phase, the preferential orientation of the grains affects the mechanical properties of the alloy. Bache and Evans [8] investigated the effect of the initial texture and the deformation behavior on the mechanical anisotropy of Ti 6/4 rolled plates and proposed that the differences in the mechanical behaviors are related to the ability to induce slips in the different plate orientations. Zhang et al. [9] found that shear bands are preferentially distributed in the γ-fiber (<111>//ND) grains in their study of the organization of the TB18 titanium alloy rolled plate.

In the field of near-β titanium alloys, many studies have focused on the texture evolution during the rolling deformation, as well as recrystallization mechanisms [10,11,12,13]. Recently, it was suggested that the effect of texture on the mechanical properties of rolled plates of near-β titanium alloys is determined by the preferential orientation of the grains parallel to the processing direction. In their study of the effect of the <110> texture on the mechanical properties of plates, Dong et al. [14] attributed the superior plasticity of near-β titanium rolled plates along the rolling direction to the fact that titanium alloy rolled plates contain more <110>//RD fiber textures with high Schmid factors.

In this paper, a new sub-stable β-Ti alloy, TB18 (Ti-5.3Cr-4.9Mo4.9V-4.3Al-0.9Nb-0.3Fe), was subjected to forging at three different levels of deformation and solid solution-aging treatments, in which the evolution of the texture is affected by the hot forging and heat treatment. In this paper, we analyze the organization evolution and mechanical properties of the alloy during the solid solution and aging processes, discuss in detail the effect of recrystallization texture on the ductility of the alloy, and finally, elucidate the relationship between microstructure, mechanical properties, and fracture mechanism. Understanding the influence of hot forging deformation on the microstructure and mechanical properties of the TB18 alloy can play a guiding role in the design of hot forging dies, as well as forgings in industrial production.

## 2. Materials and Methods

**Figure 1:**
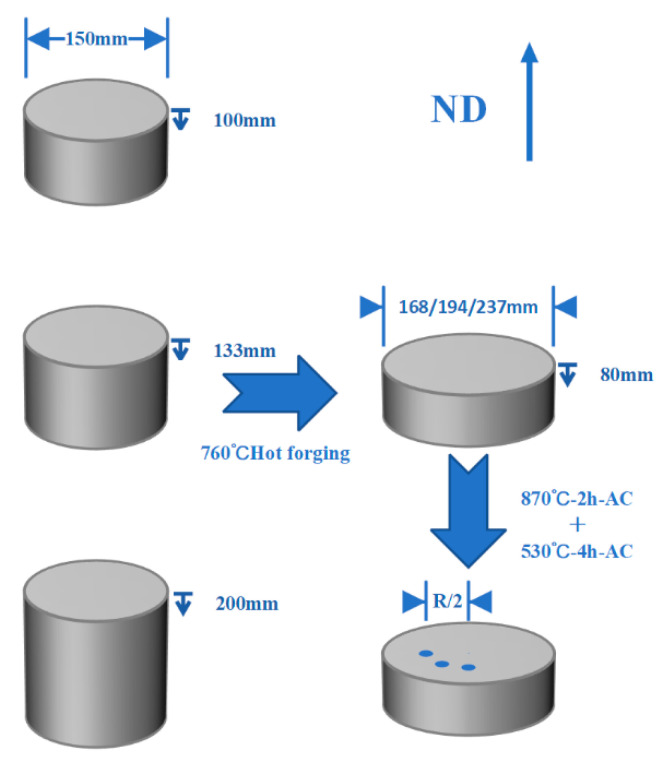
Schematic diagram of the deformation.

As shown in Figure 1, the 150 mm diameter TB 18 alloy bar was supplied by Erzhong Wanhang Die Forging Company, and the corresponding β-transition temperature was determined to be about 795 °C using the metallographic method. The as received specimens with heights of 100 mm, 133 mm and 200 mm were forged at 760 °C, and the height of the forged specimen was 80 mm, corresponding to the hot forging deformation of 20%, 40% and 60%, respectively. The hot forged TB18 specimens were subjected to solution treatment at 870 °C-2 h-Air cooling (AC) and aging treatment at 530 °C-4 h-AC, and then Φ13 × 80 mm round bars were taken from one-half of the radius of the specimen block along the machining direction of the specimen block (ND direction) by wire cutting and processed into tensile specimens. The tensile specimens were subjected to tensile testing at 20 °C using an Instron 3369 electronic universal testing machine at a strain rate of 10^−3^ s^−1^, and three tensile specimens were tested for each condition.

The samples were characterized by optical microscopy (OM) using the ZEISS Axiolab 5 after etching treatment using the Kroll reagent (2 mL HF + 6 mL HNO_3_ + 100 mL H_2_O). After the mechanical grinding and electrolytic polishing of the specimens, the samples were analyzed using Electron Backscattered Diffraction (EBSD) using an Oxford Nordly max3 field emission scanning electron microscope operated at a voltage of 20 KV with a scanning step of 2 μm, and the data were processed in OIM (ver.7.3.1 ×64) and Channel5 software (Oxford instruments HKL A/S 2007. ver.5.0.9.0). The grain orientation spread (GOS) was used to distinguish the recrystallized grains in the microstructure. The textures of the scanned regions were characterized by the orientation distribution function (ODF) over a specific section (φ2 = 45°), as calculated using a harmonic series expansion method with a maximum rank of 20. The tensile fracture was analyzed with Fhenom prox scanning electron microscopy (SEM) using a secondary electron detector (SED).

## 3. Results

### 3.1. Initial Microstructure

**Figure 2:**
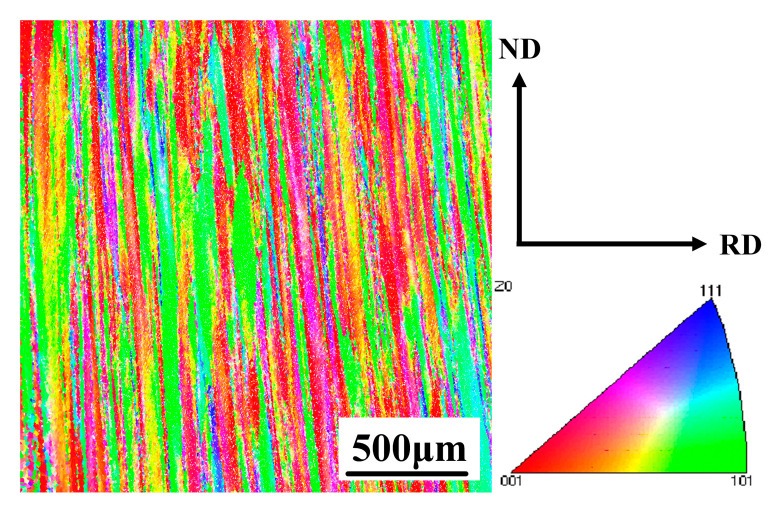
EBSD observation of the received specimen in TB18.

**Figure 3:**
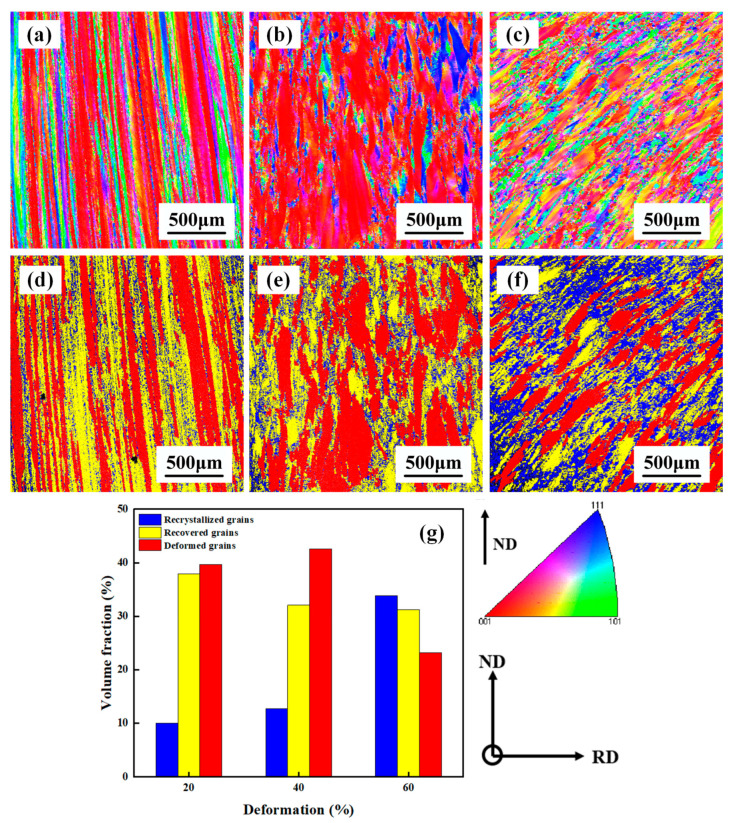
EBSD observation of the TB18 titanium alloys undergoes different levels of deformation: (**a**,**d**) ε = 0.2; (**b**,**e**) ε = 0.4; (**c**,**f**) ε = 0.6; (**g**) volume fraction of recrystallized grains.

The microstructure of the initial TB18 is shown in Figure 2. Since this experimental specimen was taken from TB18 bar stock, the external molds used during the bar-forming process implemented the extrusion of the bar along the rolling direction (RD), which made the matrix β-phase present an elongated state along the ND direction. Figure 3 shows the EBSD image of TB18 alloy after deformation at 780 °C. As shown in Figure 3a, when the deformation level is approaching 20% (ε = 0.2), the β grains do not undergo obvious plastic deformation, and the deformed grains are similar to the original grains; the β grains remain elongated and fibrous, with an average length of more than 1500 μm. The corresponding GOS map (Figure 3d) shows that the microstructure is dominated by deformed grains (41%) and recovery grains (38%), with a smaller fraction of recrystallized grains (11%). Only a small amount of dynamic recrystallization occurred during the deformation process. As shown in Figure 3b, with the increase of the deformation level to 40% (ε = 0.4), the pristine β grains were flattened, coarsened or even crushed during the forging process, and the average length was about 650 μm, at which time, the degree of dynamic recrystallization was still weak, and the content of the recrystallized grains was only 13%, as shown in Figure 3e,g. As the deformation was further increased to 60% (ε = 0.6), the level of fragmentation of the pristine β grains increased significantly, and the average length decreased to 375 μm (e.g., Figure 3c). In addition, the volume fraction of recrystallized grains increased dramatically to 34% (as shown in Figure 3f,g) as the increased deformation storage energy provided sufficient driving force for dynamic recrystallization.

**Figure 4:**
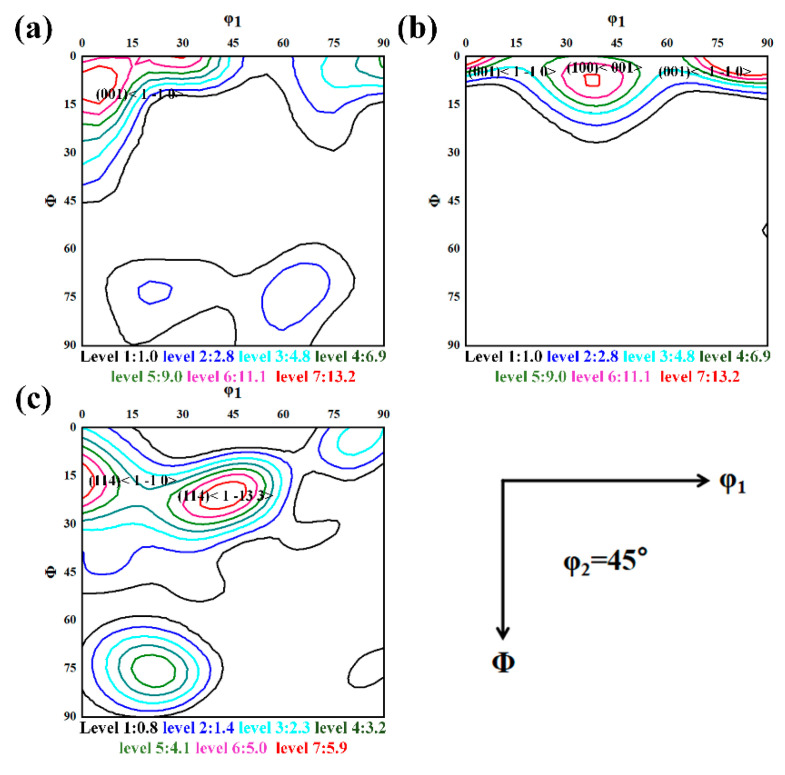
φ2 = 45° ODF section of the center area of each sample. (**a**) ε = 0.2, (**b**) ε = 0.4 and (**c**) ε = 0.2.

The texture components of the forged TB18 are characterized and shown in Figure 4. As shown in Figure 4a–c, the original samples underwent forging in the ND direction, in which the <110>//ND texture has the highest strength level. As shown in Figure 4a, the main component of the initial deformation texture at 20% deformation was the rotational cubic texture (001)[1$\bar{1}$0], which reaches a strength of 10. The strength of the rotational cubic texture (001)[1$\bar{1}$0] increased to 16.9 at 40% of the deformation when the deformed texture consists of the very strong cube cubic texture (100)<001> and the rotational cubic texture dependent on the theta texture (<001>//ND). The strength of the internal deformation texture of the sample with a hot forging deformation of 60% was weakened when the deformation texture consisted of (225)<6$\overset{-}{11}$2> and (114)<1$\;\overset{-}{13}\;$3>, with strengths of only 6.5 and 6.4, respectively. The weakening of texture strength during the increased hot forging deformation may be related to dynamic recrystallization. There have been reports that the formation of deformation texture is not only related to dislocation slip but also is closely related to the dynamic recrystallization process that occurs during thermal deformation. The recrystallized grains exhibit random orientation, weakening the deformation texture [15,16].

### 3.2. Microstructure of TB18 after Thermal Treatment

#### 3.2.1. Microstructure after Solid Solution

**Figure 5:**
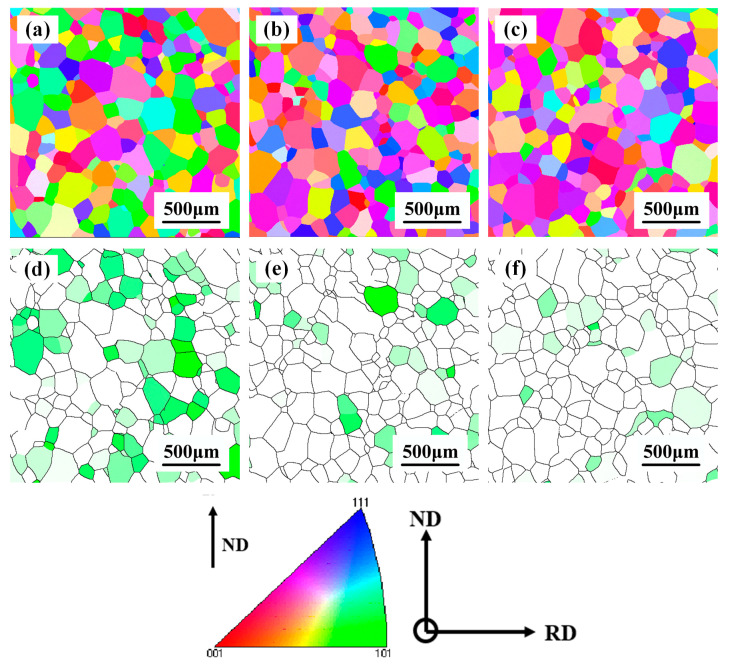
EBSD observation of TB18 specimens after solid solution. (**a**,**d**) ε = 0.2; (**b**,**e**) ε = 0.4 and (**c**,**f**) ε = 0.6.

Figure 5 shows the microstructure of the samples after the solution treatment. From Figure 5a–c, it can be seen that complete recrystallization occurred in all three groups of samples, and with the increase of deformation, the average grain size of recrystallization decreased by 5%. As shown in Figure 5d–f, as the deformation increased, the percentage of <110>//ND decreased by 12.9%, and the percentage of <100>//ND also decreased by 5.2%. Table 1 shows the statistically derived percentage of the three types of texture components, with a higher content of <110>//ND, and the total content of the three types of fiber textures decreasing progressively with increasing deformation. 

**Figure 6:**
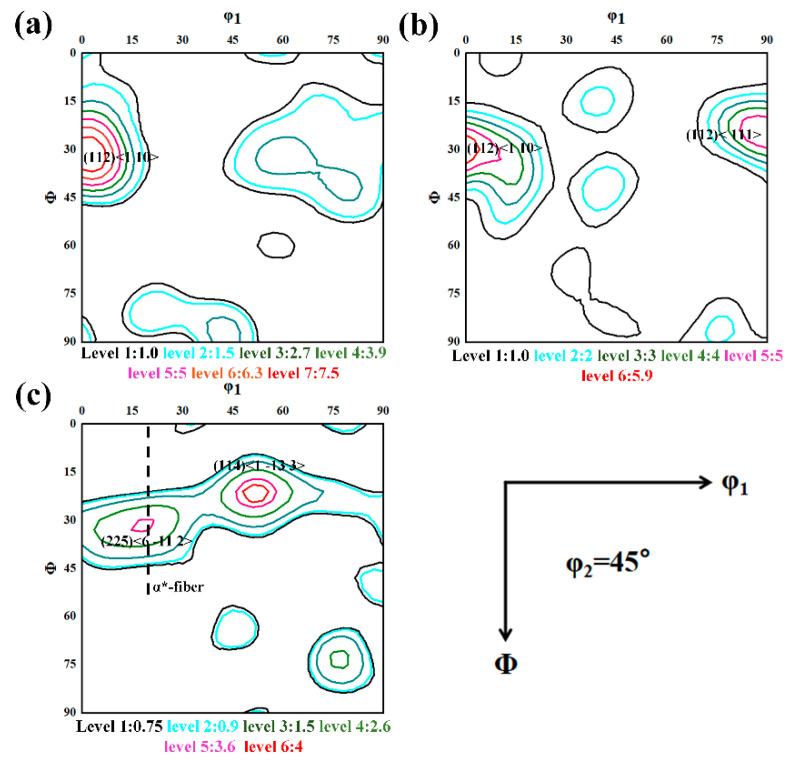
φ2 = 45° ODF section of the center area of each sample after solid solution. (**a**) ε = 0.2, (**b**) ε = 0.4 and (**c**) ε = 0.2.

The ODF diagram, corresponding to the specimen after the solid solution, is shown in Figure 6. As can be seen in Figure 6a, the strength of the dominant component (001)[1$\overset{-}{1}$0] in the deformation texture is significantly weakened after 120 min of solid solution treatment, and the main component of the texture at this time consists of the α-fiber texture (112)<110>. In Figure 6b, after the solid solution of the specimen with a deformation of 40%, all of the original rotated cube texture components and the cube texture components decomposed, and the remaining texture consisted of the weaker (112)<110> orientation, with an intensity of about 6.7. Meanwhile, the intensity of the copper texture component (112)<111> increased to 5.8 after undergoing solid solution. The α* fiber textures (225)<6$\;\overset{-}{11}$ 2> and (114)<1$\;\overset{-}{13}\;$3> fractions in Figure 6c have the highest intensities, but the intensities are only 3.6 and 4, respectively. Chen et al. reported that its formation can be attributed to recrystallization that occurs during the solid solution treatment process. The growth of recrystallized grains reduces the strength of the texture [17].

By combining the results of Figure 4 and Figure 6, two phenomena should be noted. In the first, the intensity of the original major constituents of forged TB18, after undergoing 20–40% deformation, including the rotational cubic texture (001)[1$\overset{-}{1}$0] and the cubic texture (100)<001>, was almost zero after the solid solution, suggesting that preferential recrystallization of forged TB18 occurs in a way that is dependent on the orientation of the crystals. The second is the gradual extension of the dominant component of the α-fiber texture (114) <1$\overset{-}{1}$0> in the direction of the other α-fiber textures, with the eventual texture moving toward a weak α*-fiber texture. This phenomenon has been rarely reported in titanium alloys and has been reported in steels with medium rolling regimes [18,19,20]. The evolutionary pattern of deformation texture to recrystallization texture will be explained in Section 4.2.

#### 3.2.2. Microstructure after Aging

**Figure 7:**
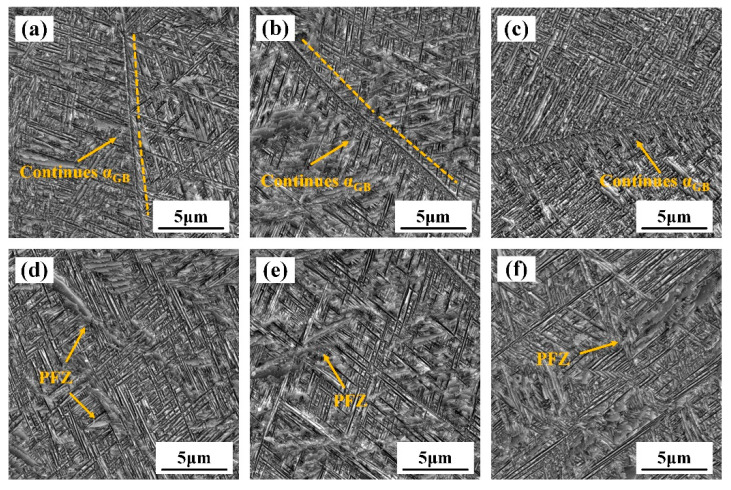
SEM observation of TB18 samples after aging. (**a**,**d**) ε = 0.2; (**b**,**e**) ε = 0.4 and (**c**,**f**) ε = 0.6.

The SEM image of the TB18 specimens, after the solid solution at 870 °C for 2 h and aging at 525 °C for 4 h, is shown in Figure 7. The boundaries in the solid solution specimens (Figure 7a–c) show continuous grain boundary α phases (αGBs); grain boundary α phases tend to be continuous thin films, and some broad grain boundary Widmannstätten α colonies (αWGBs) nucleate around the former and grow toward the interior of the grains. As shown in Figure 7d,e, the intracrystalline α-precipitates all exhibit inhomogeneous and coarser precipitation characteristics, and there are large massive precipitate-free zones (PFZs) in the β-matrix (Figure 7d,e). The average length and thickness of the α-plate within the 20% deformation specimen are approximately 1114 nm and 82 nm, respectively, and the length and thickness of the 40% deformation specimen are 1124 nm and 76 nm. At the center of the β grains of the ε = 60% specimen (Figure 7d), the average length and thickness of the α plate are 1123 nm and 78 nm, which are not different from the α phase dimensions of the specimens at the two previous deformations. Microstructural characterization showed that for the TB18 sample, increasing the initial deformation had no effect on the shape size and distribution of the α-sheets in the center of the β-grains after the solid solution and aging. There is no difference in the microstructure of TB18 alloys with different initial deformations after the same solid solution and aging treatment.

### 3.3. Mechanical Properties

Tensile tests were carried out on three parallel specimens for each sample to investigate the variation in tensile properties. Table 2 shows the tensile properties of solid-soluted and aged TB18 alloy. As shown in Table 2, when the deformation was increased from 20% to 60%, the YS and UTS of solid solution TB18 changed less, but the plasticity dropped significantly. The difference between the yield strength (YS) and ultimate tensile strength (UTS) of the aged TB18 was also minimal, but the elongation increased and then decreased with increasing deformation.

The YS, UTS and elongation (EL) of the solid soluted or aged samples are shown in Table 2, respectively, where it is observed that the deformation increased from 20% to 60%, the yield and tensile strengths of the solid soluted specimens slightly decreased, and the elongation decreased sharply with the increase in deformation. The tensile intensity and elongation of the solid soluted samples after 20% deformation could reach 821 MPa and 18.9%, respectively. With the deformation increased from 20% to 60%, the elongation reduced from 18.9% to 13.8%, which was a decrease of 27%. It can be seen that the TB18 alloy specimens with lower deformation can have better intensity and plasticity after the solid solution treatment compared with the high deformation specimens. Compared with solid soluted specimens, the elongation of the aged specimens was significantly reduced by 50%, whereas the elongation of the specimens with 40% deformation was slightly higher at 6.9%.

## 4. Discussion

### 4.1. Fracture Analysis

**Figure 8:**
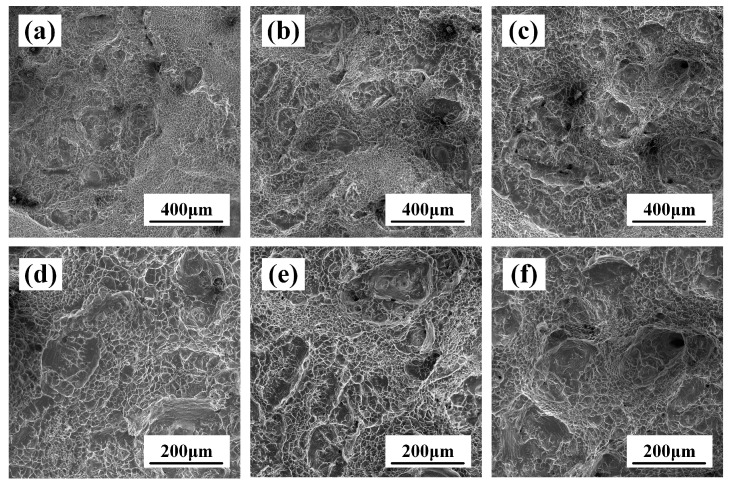
Fractography of solid-soluted TB18 alloy. (**a**,**d**) ε = 0.2, (**b**,**e**) ε = 0.4 and (**c**,**f**) ε = 0.6.

As shown in Figure 8a–c, there is a large number of “tough nests” distributed on the fracture, and the fracture mode of the tensile specimen is a tough fracture. The morphology of the ductile dimple depends on the state of stress, and during the stretching, the final shape of the ductile dimple is predominantly isometric due to normal stress [21]. In Figure 8d–f, it can be seen that there are large and small holes, as well as different sizes of equiaxial ductile dimples, distributed on the fracture, in which the average size of equiaxial ductile dimples decreases with increasing deformation, measuring 28.5 μm, 24 μm and 17.5 μm, respectively. The larger the ductile dimple size, the more severe plastic deformation occurs during the formation of the ductile dimple; then, more energy will be absorbed during the fracture process, and the fracture toughness will be higher [22]. This indicates that the larger the ductile dimple size, the higher the intensity and elongation of TB18, which is the same as our conclusion on the tensile properties in Table 2. In summary, the samples deformed with different deformation amounts fractured in the same way after the solid solution, exhibiting tough fractures.

**Figure 9:**
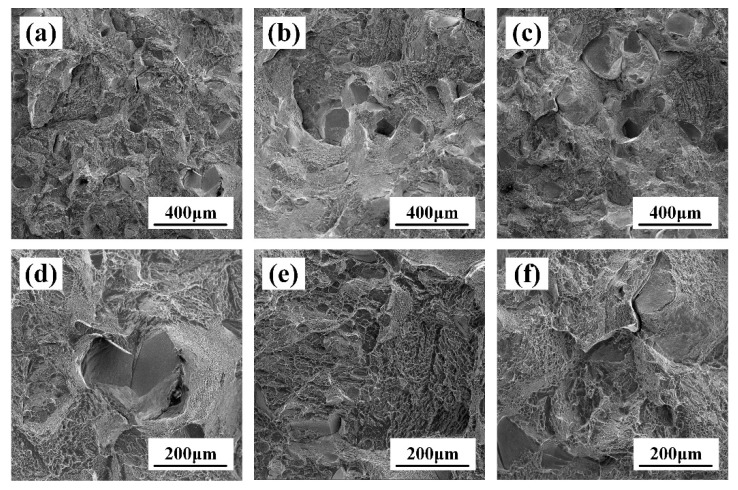
Fractography of aged TB18 alloy. (**a**,**d**) ε = 0.2, (**b**,**e**) ε = 0.4 and (**c**,**f**) ε = 0.6.

Figure 9 shows the fractography of the tensile specimen after the solid solution and aging. From Figure 9a–c, it can be seen that the fracture process of the TB18 alloy is accompanied by more plastic deformation after undergoing solid solution at 870 °C for 2 h + aging at 525 °C for 4 h, the fracture state is between the Cleavage fracture and ductile dimple fracture, which can be referred to as a quasi-Cleavage fracture. Figure 9d shows a distinct Cleavage surface as well as Cleavage steps. On Cleavage fractures of bcc metals and alloys, it is common to see raised or depressed “tongue patterns”, as in Figure 9b–d.

The fracture patterns of TB18 after the same heat treatment at different deformations are the same. Nevertheless, the difference between the fracture modes of the solid soluted tensile specimens and the aged specimens can be attributed to the continuous precipitation of the second phase during the aging process. The α-phase precipitation has an effect on the formation of microcracks and their extension paths, which results in a change in the fracture mode from ductile fracture to hybrid fracture.

**Figure 10:**
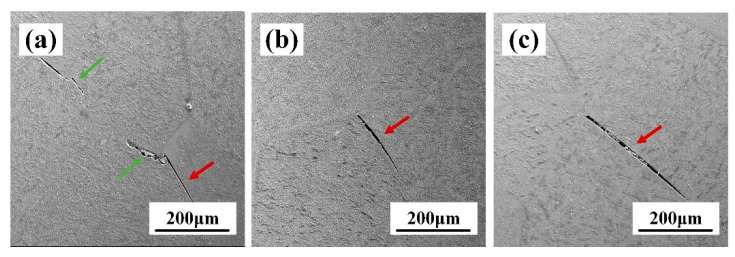
Microcrack images of TB18 alloy with different strains after aging treatment. (**a**) ε = 0.2, (**b**) ε = 0.4 and (**c**) ε = 0.6.

The central region of fracture of the tensile specimen after aging is shown in Figure 10a, with the green arrow pointing to the transgranular fracture and the red arrow pointing to the intercrystalline fracture. The specimens formed microcracks at grain boundaries under external loading, and the microcracks continued to expand forward along the lower-intensity grain boundaries, similar to the findings of Pineau et al. [23]. Caceres and Wilkinson [24] pointed out that cracks are more likely to sprout at triple-grain boundaries because of the inhibition of grain slip at the nodes of triple-grain boundaries, which leads to a higher concentration of stress and susceptibility to the formation of intercrystalline fracture. As can be seen from Figure 10b,c, for the TB18 aging specimen, the microcracks mainly sprouted at the triple grain boundaries and extended forward along the grain boundaries with lower intensity.

In short, the fracture modes of TB18 alloys with different deformations after the solid solution and aging treatment are the same and are mixed fracture modes, including ductile and brittle fractures. In this case, due to the large amount of precipitation of α-phase at grain boundaries after aging, grain slip is suppressed at the nodes of triple-grain boundaries, which promotes stress concentration, resulting in microcracks (Figure 10b). Microcracks arise and continue to widen and form voids, which tend to grow along the boundary between the two neighboring grains. Once formed, cracks continue to widen and form voids, which tend to grow along the boundary between the two neighboring grains.

### 4.2. Texture Evolution

As can be seen in Figure 3e,f and Figure 4c, dynamic recrystallization was observed for 60% deformed samples, with an overall weakening of the texture intensity. The relationship between dynamic recrystallization (DRX) and texture evolution has been widely discussed [25,26,27,28,29]. It is well known that when the grain boundaries bulge to form new DRX grains, they inherit the orientation of the neighboring matrix during subsequent deformation, thus leading to an increase in texture intensity [26]. However, the texture intensity of the specimens decreased as the amount of deformation increased in this study. It has been shown that during the hot rolling of titanium alloys, the formation of randomly distributed oriented DRX grains and the preservation of a large orientation difference between the deformed matrix and the DRX grains will lead to a weakening of the texture [27]. During the early stages of the hot forging process of titanium alloys at 1053 K, dynamically recrystallized grains with similar orientations are more inclined to occur along the grain boundaries. When deformation is increased, those formed DRX grains start to rotate toward the preferred slip system [30,31,32,33,34]. Due to the presence of multiple slip systems in β-titanium alloys and the presence of inherently large orientation differences between the slip systems [34], these DRX grains retain large orientation differences after large deformations, which provide random orientations to these DRX grains, ultimately weakening the intensity of the deformed texture in TB18 alloys.

As can be seen from Figure 4, when the hot forging is at 60% deformation, the deformation texture intensity in the samples increases with the increase of deformation in the early stage of deformation, and when 20% deformation is reached, new DRX grains are formed at the grain boundaries of the crushed β-deformed grains due to the initiation of the dynamic recrystallization, and the formation of these randomly distributed oriented DRX grains ultimately leads to the weakening of the deformation texture intensity.

Figure 4 and Figure 6 point out the type of texture of TB18 titanium alloy before and after the solid solution. In the 20% versus 40% deformation samples, the main pattern of texture evolution is that the main component of the texture deviates from the rotated cube texture to the α-fiber texture, as well as to the copper texture after the solid solution treatment and, at the same time, the cube texture component undergoes decomposition. Figure 6b shows that the type of texture formed after deviation of the rotated cube texture with a larger φ1 angle is copper texture. When the deformation is increased to 60%, the deformed texture is similar to the post-solid soluted texture characteristics, the orientation does not undergo a major deviation, and the main composition is dominated by the α-fiber texture with the (114)<1$\;\overset{-}{13}\;$3> texture. The probability of transformation into recrystallized grains also increases with an increase in the mean orientation difference angle, which is in agreement with the common view [33,34]. 

**Figure 11:**
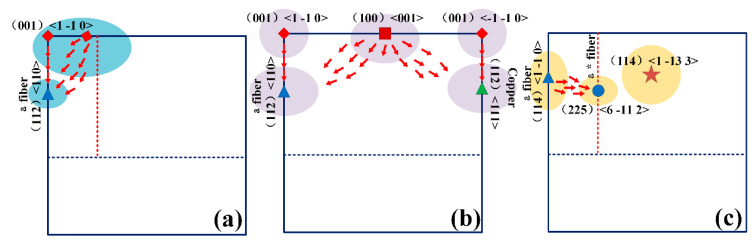
Schematic diagram of texture evolution. (**a**) ε = 0.2; (**b**) ε = 0.4; (**c**) ε = 0.6.

The article [35] notes that the subgrains of α-fibers with (001)<110>, (112)<110> and (111)<110> orientations do not exhibit particularly extreme boundary properties and that the above orientations also constitute some of the major components of the recrystallization texture. Thus, the presence of such selective orientations in the recrystallized state can be attributed to their high-volume fraction in the deformed state. In contrast, subgrains with the cub texture orientation (001)<100> have higher boundary energies and mobility, which makes them more likely to become recrystallized grains, as does the evolution of rotated cube texture. As a result, some texture components preferentially decompose during recrystallization, as shown in the results of this paper (Figure 4b and Figure 6b). The specific texture evolution is shown schematically in Figure 11.

In summary, the texture evolution of the TB18 alloy at low deformation (20–40%) is characterized by a weakening of the rotated cube texture that deviates into an α-fiber texture, which is accompanied by a complete disintegration of the cube texture orientation. The mechanism of texture evolution is that the rapid growth of minor components during the recrystallization weakens the intensity of the deformed texture, followed by the higher boundary energy and mobility of subgrains of cubic orientation (001)<100>, which makes them more likely to turn into recrystallized grains, with the preferential decomposition of the cube texture components and a decrease in the intensity of the deformation texture. The α-fiber orientation, on the other hand, will not exhibit particularly extreme boundary properties and will be retained as a component of the recrystallized texture. At high deformation (60%), as the initial deformation texture of the alloy is affected by dynamic recrystallization during the hot forging, the main components are α-fiber texture with (114)<1$\;\overset{-}{13}\;$3> texture. After subsequent static recrystallization, a deflection from α-fiber texture to α*-fiber texture occurs, which is similar to the findings of Zhang et al. [9]. The (114)<1$\;\overset{-}{13}\;$3> texture, on the other hand, may be inherited as a major component of the recrystallization texture even after a solid solution due to its lower boundary energy and mobility.

### 4.3. Effect of Texture on Mechanical Properties

An increase in the amount of deformation leads to an increase in the deformation storage energy of the alloy [36]. As shown by the EBSD observations in Figure 5, the increased deformation contributed to the refinement of the alloy grains, although this dimensional change was not obvious. Interface strengthening stands out as a primary method for enhancing the strength of alloys [37]. Grain refinement possesses the capability to elevate the quantity of interfaces, subsequently augmenting plasticity. Nevertheless, as depicted in Table 2, the average grain size decreases while the plasticity of the TB18 alloy diminishes. Figure 8, Figure 9 and Figure 10 illustrate that all three deformation samples exhibited identical fracture behavior post the solid solution, characterized by ductile fracture. Following the solid solution-aging treatment of the specimens under different levels of deformation, it is apparent that there are no discernible variations in the microstructure, with the fracture mode remaining consistent. Specifically, a mixed fracture mode is observed, encompassing both ductile and brittle fracture components. The aforementioned evidence demonstrates that the tensile characteristics of the alloy in the ND orientation cannot be solely explained by grain boundary strengthening. Therefore, an examination of the main issue of alloy texture might offer clarity to the uncertainties at hand.

In Table 1, the data indicate a gradual decrease in the proportion of different textures in TB18 as the amount of deformation increases. When conducting texture analysis on near-β titanium alloy hot-rolled sheets, Dong et al. [14] asserted that the impact of texture on the mechanical characteristics of the alloy correlates with the favored orientation of grains aligning parallel to the processing direction. The elevated volume fraction of <110> fiber texture serves as the primary factor contributing to its decreased strength and heightened plasticity. It has also been reported that the mechanical properties of the TB18 alloy in the ND direction are related to the distribution of textures parallel to the ND direction [14]. Table 1 illustrates that the presence of the <110>//ND texture in the specimen subjected to 20% deformation exceeds that of the samples deformed by 40% and 60%. Correspondingly, the amount of this texture in the material deformed at 40% surpasses that of the specimen deformed at 60%. The relationship demonstrates that as the deformation percentage increases, the levels of this texture follow a similar pattern to the elongation behavior of TB18 post-solid solution, as outlined in this study. It can be generalized that the large presence of the <110>//ND texture in the solid-soluted TB18 with deformation of 20% is the main reason for its optimal plasticity.

By the Burgers relationship, (0001)_α_//(110)_β_, and [11$\overset{-}{2}$0]_α_//[1$\overset{-}{1}$1]_β_. The alloy structure after aging consists of α texture and β texture, as shown in Table 1. The content of the <111>//ND texture in the alloy with a deformation of 40% was greater than 20%, and it was several times that of the latter. The α phase texture corresponding to the <111>//ND texture was [11$\overset{-}{2}$0]_α_//ND. At the same time, the content of the <110>//ND texture in the alloy with a deformation of 20% was greater than 40%, and the α phase texture corresponding to the <110>//ND texture was [0001]_α_//ND. Each texture’s Schmid factor calculation results are shown in Table 3. [11$\overset{-}{2}$0]_α_//ND has a higher SF value, which greatly improves the plasticity of the alloy. The basal texture fiber texture [0001]_α_//ND is detrimental to ductility. The strengthening of the basal texture weakens the fracture strain in the tension and compression of the alloy [38].

In short, in the stretching direction ND, the contribution of the β phase texture to the alloy plasticity mainly depends on the presence of the <110>//ND texture. The SF value of [11$\overset{-}{2}$0]_α_//ND in the α phase texture is relatively high, so the content of this texture can also affect the alloy plasticity. In the TB18 alloy with different deformation amounts, the performance law caused by the β texture is that the elongation decreases with the increase in deformation amount. After the aging, due to the Burgers orientation relationship, the alloy with 40% deformation has a higher content of [111]_β_ texture than the alloy with 20% deformation, and the [11$\overset{-}{2}$0]_α_//ND content formed during aging is more than that of the alloy with 20% deformation, while the [0001]_α_//ND content inherited from <110>//ND is less than that of the alloy with 20% deformation. The high SF value of [11$\overset{-}{2}$0]_α_//ND can improve the plasticity of the alloy, while the strengthening of the base texture [0001]_α_//ND will lead to a decrease in the elongation of the alloy. On the whole, the combined effect of α texture and β texture results in the decreasing elongation after aging for the TB18 alloy, with the sequence as follows: samples with deformation of 40% > samples with deformation of 20% > samples with deformation of 60%.

## 5. Conclusions

This article investigates the microstructural evolution of the TB18 alloy during the solid solution process at 1143 K, as well as the mechanical properties of the alloy along the normal direction (ND) after solid solution. The following conclusions can be drawn:

(1) The texture evolution law of TB18 alloy under low deformation (20–40%) is the rotation cube texture offset to the α fiber texture, accompanied by the complete decomposition of the cube texture orientation. Under high deformation (60%), the main components of the deformed texture are the α fiber texture and the specific orientation texture (114)<1$\;\overset{-}{13}\;$3>. After subsequent static recrystallization, the α fiber texture deviates to the α* fiber texture, while the specific orientation (114)<1$\;\overset{-}{13}\;$3> can still be inherited as the main component of the recrystallization texture.

(2) The plasticity of the alloy in the ND direction after the solid solution is greatly affected by the content of the <110>//ND texture. The content of the <110>//ND texture in the matrix after aging and in the α precipitated phase [11$\overset{-}{2}$0]_α_//ND has a significant impact on the plasticity of the alloy in the ND direction. In the ND direction, as the initial deformation amount increases, the elongation of the alloy shows a decreasing trend after the solid solution. Compared with the solid solution state sample, the elongation of the aged sample is significantly reduced, and the elongation of the sample with a deformation amount of 40% is slightly higher, which is mainly due to the combined influence of α texture and β texture.

## Figures and Tables

**Table 1:** Volume fraction of texture after solid solution along ND direction, %.

Fiber Texture	ε = 0.2	ε = 0.4	ε = 0.6
<111>//ND	9.89	16.10	8.42
<110>//ND	37.81	18.51	14.9
<100>//ND	13.70	13.80	8.48

**Table 2:** Tensile properties of solid-soluted or aged TB18 alloys.

Treatment	Strain	Yield Strength/MPa	Tensile Strength/MPa	Elongation/%
Solution	0.2	817 ± 4	821 ± 6	18.9 ± 0.8
Solution	0.4	811 ± 8	814 ± 5	17.3 ± 0.7
Solution	0.6	810 ± 7	810 ± 5	13.8 ± 1.1
Solution + aging	0.2	1275 ± 12	1356 ± 13	6.4 ± 0.6
Solution + aging	0.4	1265 ± 12	1344 ± 14	6.9 ± 0.6
Solution + aging	0.6	1265 ± 18	1338 ± 12	6.2 ± 0.6

**Table 3:** hcp texture and their Schmid factor.

Texture	Dominant Slip Mode	SF
$(11\overset{-}{2}$$0)[1\overset{-}{1}$00]	$\mathrm{Pyramidal}\text{<}a\text{>}\mathrm{slip}(\overset{-}{1}$$011)[1\overset{-}{2}$10]	0.439
$(02\overset{-}{2}$$3)[\overset{-}{2}$110]	$\mathrm{Basal}\text{<}a\text{>}\mathrm{slip}(0001)[1\overset{-}{2}$10]	0.462
$(11\overset{-}{2}$$0)[1\overset{-}{1}$00]	$\mathrm{pyramidal}\text{<}a\text{>}\mathrm{slip}(10\overset{-}{1}$$1)[\overset{-}{1}$$2\overset{-}{1}$0]	0.439
$(0001)[1\overset{-}{1}$00]		0

## Data Availability

The authors confirm that the data supporting the findings of this study are available within the article.
